# Public awareness of advance care planning and hospice palliative care: a nationwide cross-sectional study in Korea

**DOI:** 10.1186/s12904-023-01333-y

**Published:** 2023-12-27

**Authors:** Boram Kim, Junyong Lee, Youn Seon Choi

**Affiliations:** 1 Division of Health Policy, Bureau of Health Policy, Ministry of Health and Welfare, Sejong-si, Republic of Korea; 2 Department of Family Medicine, Veterans Health Service (VHS) Medical Center, Seoul, Republic of Korea; 3 Department of Family Medicine, Korea University Guro Hospital, Seoul, Republic of Korea

**Keywords:** Advance care planning, Hospice palliative care, Health care experience, Awareness

## Abstract

**Context:**

Advance care planning (ACP) and hospice palliative care (HPC) have potential benefits for individuals and health systems. Public awareness of them might increase their acceptance.

**Objectives:**

To examine public awareness of ACP and HPC and related factors including individuals’ experience of health care among Korean population.

**Methods:**

A cross-sectional study based on a nationally representative sample was conducted. Data from participants aged 15 years or older were examined. Socio-demographic characteristics, health-related factors, health care experience in the past year, and awareness of ACP and HPC were analyzed. Subgroup analysis was conducted to determine associations between specific experiences during outpatient visit and awareness of ACP and HPC.

**Results:**

Of a total of 13,546 subjects, 39.3% and 35.7% reported awareness of ACP and HPC, respectively. About half (48.6%) of participants reported that they were completely unaware of ACP or HPC. Recent outpatient visit was positively associated with HPC awareness. Participants were more likely to recognize ACP or HPC if they had experience in hospitalization and health checkup over the past year and had trust in the medical system. Conversely, participants who had inadequate health care access due to cost burden showed low awareness of ACP and HPC.

**Conclusion:**

There was a lack of public awareness of ACP and HPC. There were significant differences depending on various factors, especially individual health care experiences. Appropriate interventions are needed to facilitate discussion of ACP and HPC, thereby increasing public awareness.

## Introduction

Advance care planning (ACP) is a process that encourages adults of all ages with various health status to understand and share personal values, life goals, and preferences for future health care [1]. Implementation of ACP has potential benefits for individuals, their families and friends, and the health care system. ACP can promote individuals’ autonomy by facilitating the planning of end-of-life care in preparation for a loss of decision-making capacity [2]. It can also improve the quality of patient-physician communication [3]. Furthermore, ACP can reduce the burden of family and friends as surrogate decision makers for patients [4]. It can also decrease the use of health care resources for unnecessary hospitalization and life-sustaining treatment [2, 3]. Hospice palliative care (HPC) is defined as ‘an active and holistic treatment for individuals of all ages, especially those near death, who are experiencing significant health-related distress as a result of a serious illness’ [5]. It aims to address physical, psychological, and spiritual needs, thus improving the quality of life for patients, their families, and caregivers [5, 6]. HPC is associated with improved patient symptom control, increased satisfaction with care, and reduced hospitalizations or emergency department visits [7, 8]. Despite these benefits of ACP and HPC, both remain underutilized by the public, showing significant socioeconomic, racial, and national disparities in utilization [6, 9–13].

Several factors contribute to the under-utilization of ACP and HPC, one of which is the lack of public awareness of these services [10, 11, 14–16]. Although putting knowledge into action is challenging, several existing studies have shown that facilitating conversation and improving awareness can lead to positive changes in preference or implementation of ACP and HPC [17, 18].

Moreover, as the world faces an aging population and an increasing in number of patients with chronic and incurable diseases, strategies to increase awareness and promote the use of ACP and HPC are becoming important for improving quality of care [19, 20]. The percentage of people aged 65 years or more of the world is projected to increase from 9.8% in 2022 to 20.1% in 2070 [21]. That percentage in Korea is projected to increase from 17.5% in 2022 to 46.4% in 2070 [21]. The number of deaths in Korea was about 310,000 in 2020. It is expected to continue to rise to about 700,000 in 2070 [22]. Serious illness such as cancer and heart disease are leading causes of death in Korea [23], suggesting that the demand for high-quality ACP and HPC will increase.

Although HPC was introduced in Korea in the 1960s, national policies to support HPC did not begin until the 2000s [24, 25]. The government announced the standard for official designation of HPC units in 2008. Inpatients have been covered by the National Health Insurance since 2015 [24, 26]. As of 2022, there are about 180 designated institutions throughout Korea, providing care by operating beds for inpatients or home-based service. The annual number of cancer patients who used designated HPC units accounted for about 23% of all cancer deaths in 2021 [27]. The Korean government’s policy efforts on the ACP began in the 2010s. Laws to regulate it were newly enacted by merging with the existing law on HPC in 2016. The “Act on hospice and palliative care and decisions on life-sustaining treatment for patients at the end of life” has been enforced since 2018. It includes advance directives (AD) and physician orders for life-sustaining treatment (POLST) as legal forms of ACP [28]. In addition, the National Health Insurance began providing insurance coverage for ACP counseling and documentation for terminally ill patients from the enforcement of the Act. As of 2022, there are 612 registry agencies providing counseling and registering AD as a legal document and 371 government-registered medical institutions implementing ACP [29]. As a result of such changes, Korea was ranked 32nd out of 40 countries in 2010 in the Quality of Death Index (QDI) conducted by the Economist Intelligence Unit [25]. In 2015, Korea showed an improved result, ranking 18th out of 80 countries [25].

However, most Korean physicians still experience serious difficulties in communicating with patients and families about end of life (EOL) care [30]. In Korea, patients are referred for HPC very late during the course of a disease [31]. Considering that previous studies have reported that sufficient awareness of ACP and HPC is related to service acceptability, it is important to assess how aware the public is of ACP and HPC [17, 18, 20]. In addition, awareness-related factors need to be explored to develop effective strategies to increase public awareness of ACP and HPC by facilitating discussion and preparation. To date, studies on awareness and related factors related to ACP and HPC in Korean population are lacking. In particular, there have been no studies examining awareness based on national data after enaction of new laws on ACP and HPC. Thus, this study aimed to investigate the awareness of ACP and HPC in community-dwelling Korean and to explore associated variables including sociodemographic factors, health-related factors, and experience of health care as influencing factors.

## Methods

### Study design and data collection

This was a cross-sectional study using data from the 2021 Medical Service Experience Survey conducted by the Korea Institute for Health and Social Affairs at the request of the Ministry of Health and Welfare, Republic of Korea. The nationwide survey has been conducted every year since 2017 to investigate how Koreans experience services of medical institutions and how they evaluate the health care system. Based on survey district of the Population and Housing Census, 6,000 households were selected as a sample. After determining the national sample size as 600 survey districts, sample design was carried out for 26 regional layers based on the number of households in the survey population distribution. The survey was conducted between July and September 2021. Surveyors visited households and conducted interviews with household members aged 15 years or older using structured questionnaires. Targeted households were visited by surveyors at least three times, but when the survey could not be conducted due to reasons such as being unable to meet or refusing to respond, the household subject was replaced. The survey methodology has been described elsewhere [32, 33].

This study was approved by the Institutional Review Board (IRB) of Veterans Health Service Medical Center in Korea (IRB approval number: BOHUN 2023-06-013; date of approval: 4 July 2023).

### Measurements

We analyzed sociodemographic characteristics, health status, health insurance, experience of health care, trust in the health care system, and awareness of ACP and HPC of participants. Sociodemographic characteristics included age, gender, education level, and household income. Chronic health conditions were defined as having been treated in the past year for any of the following: hypertension, diabetes, mental and behavioral disorders, respiratory diseases, cardiovascular diseases, cerebrovascular diseases, disease of the nervous system, cancer, thyroid diseases, hepatic diseases, chronic renal failure, or any other chronic diseases. To ascertain participants’ health insurance, the type of public health insurance and purchase of supplemental private health insurance were investigated. Participants responded whether they had outpatient visit, had been hospitalized, had health checkups, and had any experience of giving up health care services due to financial reason in the past year. Medical use for health checkup, cosmetic improvement, and obesity treatment were not included in outpatient visit or hospitalization experiences. Participants who had an outpatient visit were asked whether the medical institution was a regular source of care, whether they were able to have a sufficient conversation with the physician during the visit, and whether the physician made an effort to make decisions together with the participant about examination or treatment. Regular source of care was asked as a dichotomous choice (yes or no). Having sufficient conversation and involving in decisions about care and treatment were measured on a five-point scale and categorized as “Yes” (“strongly agree” and “agree”) or “No” (“neutral”, “disagree” and “strongly disagree”). We investigated whether participants had experienced any national health screening program, workplace health checkups, or self-funded health checkups. Participants were asked whether they could not visit a medical institution, undergo necessary examinations or treatment, had not been prescribed, or purchased necessary medicines because of the burden of medical expenses. We defined participants as having inadequate access to health care due to cost burden if they had at least one of these experiences. Participants answered whether they had trust in the health care system. The response was measured on a five-point scale and categorized as “Trust” (“strongly agree” and “agree”) or “Distrust” (“neutral”, “disagree” and “strongly disagree”). Awareness of ACP and HPC was measured after providing brief definitions to facilitate participants’ understanding [32]. ACP was informed to participants as “a process to decide on withholding or withdrawing life-sustaining treatment such as CPR and ventilator in preparation for the EOL”. HPC was informed to participants as “a health care service to provide comprehensive care, including pain and symptom relief, for patients with serious illnesses and their families”. Participants rated their ACP and HPC awareness on a 5-point scale, respectively. Responses of “strongly agree” and “agree” were classified as “Yes” and responses of “neutral”, “disagree” and “strongly disagree” were classified as “No”.

### Statistical analysis

Data were analyzed using weighting factors based on the complex sample design of the survey. Baseline characteristics of all participants are expressed as numbers (unweighted) or percentages and standard errors (weighted). An analysis was also conducted to determine the distribution of participants’ awareness of ACP and HPC. We conducted a chi-square test to determine the relationship between variables and awareness of ACP or HPC. We conducted multivariable analysis to investigate influencing factors related to awareness of HPC and ACP, including variables identified as relevant in univariate analysis. Multivariable logistic regression was also performed to determine associations between specific experiences during outpatient visit and awareness of ACP or HPC. Variables identified by univariate analysis to be associated with awareness of ACP or HPC were included in multivariable logistic regression analysis. We used multivariable logistic regression models to estimate adjusted odds ratios (AORs) and 95% confidence intervals (CI). All statistical analyses were performed IBM SPSS Statistics 28 (SPSS Inc., Chicago, IL, USA).

## Results

### Study population

Characteristics of the 13,546 participants are presented in Table 1. Females accounted for about half (50.1%) of all participants. Regarding age, those aged 60 years or more accounted for 28.2%, higher than percentages of other age groups. The majority (95.3%) of participants had received 7 or more years of education. Less than a quarter of participants had one or more chronic diseases (23.5%) and perceived their health status as poor (20.5%). Most (97.8%) participants were covered by the National Health Insurance. The majority (73.9%) of participants had supplemental private health insurance. More than half (54.1%) of participants had outpatient visit. However, few (1.6%) participants had experienced hospitalization in the past year. Less than half (40.7%) of participants had ever had a health checkup. Some (7.6%) participants responded that they experienced inadequate access to health care due to cost burden in the past year. The majority (67.4%) of participants had trust in the health care system. Of all participants, 39.3% and 35.7% responded that they were aware of ACP and HPC, respectively.

Additional survey results from 7,782 participants who had outpatient visits were as follows (tabular data not shown): 87.8% regarded medical institution as a regular source of care; 81.4% had sufficient conversation with physician; and 89.2% were involved in decisions about their care and treatment.

**Table 1:** Baseline characteristics of study subjects (n = 13,546, N = 45.7)

Factors	Total		
	n	N	% (SE)
Gender			
Male	6,447	22.8	49.9 (0.5)
Female	7,099	22.8	50.1 (0.5)
Age (years)			
15–19	567	2.1	4.6 (0.2)
20–29	1,872	7.2	15.7 (0.4)
30–39	1,865	7.8	17.1 (0.4)
40–49	1,847	7.4	16.3 (0.4)
50–59	3,172	8.3	18.1 (0.3)
≥ 60	4,223	12.9	28.2 (0.4)
Education (years)			
≤ 6	701	2.2	4.7 (0.2)
7–12	7,221	22.0	48.1 (0.5)
≥ 13	5,624	21.5	47.1 (0.5)
Household income quintile			
1st (lowest)	2,704	8.5	18.5 (0.4)
2nd	2,829	9.2	20.1 (0.4)
3rd	2,634	9.5	20.7 (0.4)
4th	2,761	9.5	20.9 (0.4)
5th (highest)	2,618	9.0	19.8 (0.4)
Chronic health conditions			
No	9,989	34.9	76.5 (0.4)
Yes	3,557	10.7	23.5 (0.4)
Self-rated health			
Good	10,237	36.3	79.5 (0.4)
Poor	3,309	9.4	20.5 (0.4)
Type of public health insurance			
National Health Insurance	13,227	44.6	97.8 (0.1)
Medical Aid	319	1.0	2.2 (0.1)
Purchase of supplemental private health insurance			
No	3,594	11.9	26.1 (0.4)
Yes	9,952	33.7	73.9 (0.4)
Recent outpatient visit			
No	5,764	21.0	45.9 (0.5)
Yes	7,782	24.7	54.1 (0.5)
Hospitalization in the past year			
No	13,329	45.0	98.4 (0.1)
Yes	217	0.7	1.6 (0.1)
Health checkup in the past year			
No	7,702	27.0	59.3 (0.5)
Yes	5,844	18.6	40.7 (0.5)
Inadequate access to health care due to cost burden in the past year			
No	12,309	42.2	92.4 (0.2)
Yes	1,237	3.5	7.6 (0.2)
Trust in health care system			
Distrust	4,319	14.9	32.6 (0.5)
Trust	9,227	30.8	67.4 (0.5)
Awareness of advance care planning			
No	8,562	27.7	60.7 (0.5)
Yes	4,984	18.0	39.3 (0.5)
Awareness of hospice-palliative care			
No	8,870	29.4	64.3 (0.5)
Yes	4,676	16.3	35.7 (0.5)

Percentages were weighted to yield nationally representative estimates

n, unweighted sample size; N, weighted sample size in millions; SE, standard error

### Distribution of participants’ awareness of ACP and HPC

**Fig. 1:**
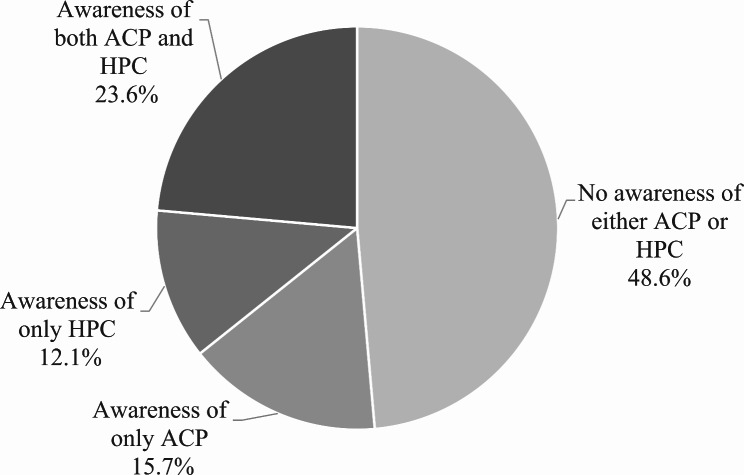
Distribution of awareness of advance care planning and hospice palliative care (n = 13,546, N = 45.7) Percentages were weighted to yield nationally representative estimates n, unweighted sample size; N, weighted sample size in millions

Distribution of participants’ awareness of ACP and HPC is shown in Fig. 1. About half (48.6%) of participants stated that they were unaware of either ACP or HPC. Among all participants, 15.7% responded that they were only aware of ACP and 12.1% responded that they only knew HPC. Those who responded that they knew both ACP and HPC accounted for 23.6% of total participants.

### Factors associated with awareness of ACP and HPC

Table 2 shows results of univariate analysis for factors related to ACP and HPC awareness. Participants aged 20 years or older, those who had 7 or more years of education, those who had household income between second and fourth quintiles, those who reported self-rated health as good, those who had experience of hospitalization and health checkup in the past year, those who reported no inadequate access to health care due to cost burden, and those who had trust in health care system compared those who did not have higher awareness of ACP and HCP. Males had higher ACP awareness then females. Participants who had supplemental private health insurance and had experience of recent outpatient visit showed higher HPC awareness than those who did not have. There was no significant difference in ACP awareness according to chronic health conditions, type of public health insurance, purchase of supplemental private health insurance, or outpatient visit. In addition, there was no significant difference in HPC according to gender, chronic health condition, or type of public health insurance.

**Table 2:** Factors associated with awareness of advance care planning and hospice palliative care by univariate analysis (n = 13,546, N = 45.7)

Factors	Advance care planning			Hospice palliative care		
	No	Yes	*p* value^a^	No	Yes	*p* value^a^
	n (%)	n (%)		n(%)	n (%)	
Gender						
Male	3,986 (59.4)	2,461 (40.6)	0.010	4,169 (63.7)	2,278 (36.3)	0.213
Female	4,576 (61.9)	2,523 (38.1)		4,701 (64.9)	2,398 (35.1)	
Age (years)						
15–19	430 (73.3)	137 (26.7)	< 0.001	427 (76.2)	140 (23.8)	< 0.001
20–29	1,346 (71.0)	526 (29.0)		1,339 (71.3)	533 (28.7)	
30–39	1,131 (58.9)	734 (41.1)		1,198 (63.0)	667 (37.0)	
40–49	1,050 (55.8)	797 (44.2)		1,113 (59.1)	734 (40.9)	
50–59	1,858 (53.9)	1,314 (46.1)		1,963 (59.7)	1,209 (40.3)	
≥ 60	2,747 (61.2)	1,476 (38.8)		2,830 (65.3)	1,393 (34.7)	
Education						
≤ 6	500 (69.0)	201 (31.0)	< 0.001	537 (76.9)	164 (23.1)	< 0.001
7–12	4,598 (60.7)	2,623 (39.3)		4,766 (65.0)	2,455 (35.0)	
≥ 13	3,464 (59.8)	2,160 (40.2)		3,567 (62.4)	2,057 (37.6)	
Household income quintile						
1st (lowest)	1,783 (62.6)	921 (37.4)	< 0.001	1,842 (66.8)	862 (33.2)	< 0.001
2nd	1,711 (58.4)	1,118 (41.6)		1,704 (60.6)	1,125 (39.4)	
3rd	1,507 (55.6)	1,127 (44.4)		1,557 (58.7)	1,077 (41.3)	
4th	1,711 (59.0)	1,050 (41.0)		1,814 (63.3)	947 (36.7)	
5th (highest)	1,850 (68.3)	768 (31.7)		1,953 (72.8)	665 (27.2)	
Chronic health conditions						
No	6,259 (60.5)	3,730 (39.5)	0.417	6,490 (64.1)	3,499 (35.9)	0.420
Yes	2,303 (61.4)	1,254 (38.6)		2,380 (65.0)	1,177 (35.0)	
Self-rated health						
Good	6,209 (59.0)	4,028 (41.0)	< 0.001	6,500 (62.7)	3,737 (37.3)	< 0.001
Poor	2,353 (67.1)	956 (32.9)		2,370 (70.7)	939 (29.3)	
Type of public health insurance						
National Health Insurance	8,360 (60.6)	4,867 (39.4)	0.504	8,650 (64.2)	4,577 (35.8)	0.215
Medical Aid	202 (62.7)	117 (37.3)		220 (68.0)	99 (32.0)	
Purchase of supplemental private health insurance						
No	2,332 (61.7)	1,262 (38.3)	0.209	2,423 (66.3)	1,171 (33.7)	0.016
Yes	6,230 (60.3)	3,722 (39.7)		6,447 (63.6)	3,505 (36.4)	
Recent outpatient visit						
No	3,705 (60.9)	2,059 (39.1)	0.693	3,955 (66.9)	1,809 (33.1)	< 0.001
Yes	4,857 (60.5)	2,925 (39.5)		4,915 (62.1)	2,867 (37.9)	
Hospitalization in the past year						
No	8,446 (60.9)	4,883 (39.1)	0.005	8,745 (64.4)	4,584 (35.6)	0.050
Yes	116 (50.2)	101 (49.8)		125 (57.1)	92 (42.9)	
Health checkup in the past year						
No	5,060 (63.0)	2,642 (37.0)	< 0.001	5,167 (66.2)	2,535 (33.8)	< 0.001
Yes	3,502 (57.3)	2,342 (42.7)		3,703 (61.6)	2,141 (38.4)	
Inadequate access to health care due to cost burden in the past year						
No	7,586 (59.4)	4,723 (40.6)	< 0.001	7,896 (63.2)	4,413 (36.8)	< 0.001
Yes	976 (76.8)	261 (23.2)		974 (77.3)	263 (22.7)	
Trust in health care system						
Distrust	3,152 (70.9)	1,167 (29.1)	< 0.001	3,136 (71.1)	1,183 (28.9)	< 0.001
Trust	5,410 (55.7)	3,817 (44.3)		5,734 (61.1)	3,493 (38.9)	

Percentages were weighted to yield nationally representative estimates

n, unweighted sample size; N, weighted sample size in millions

^a^Chi-square test was used

Results of multivariable logistic regression analysis on factors related to ACP and HPC awareness are presented in Table 3. The following variables were included in the analysis: gender, age, education, household income, self-rated health, purchase of supplemental private health insurance, experience of recent outpatient visit, experience of hospitalization and health checkup, inadequate access to health care due to cost burden, and trust in health care system. After controlling for all other variables in the model, the awareness of either ACP or HPC was significantly associated with age, education, household income, self-rated health, experience of hospitalization and health checkup, inadequate access to health care due to cost burden, and trust in health care system. Females had lower odds of being aware of ACP (AOR = 0.92, 95% CI = 0.84–1.00), whereas gender had no significant association with HPC awareness. Compared with those aged 15–19 years, individuals aged 30–39 (AOR = 1.76, 95% CI = 1.35–2.30), 40–49 (AOR = 1.97, 95% CI = 1.51–2.55), 50–59 (AOR = 2.31, 95% CI = 1.80–2.95), and ≥ 60 years (AOR = 1.99, 95% CI = 1.55–2.55) had greater odds of being aware of ACP. Similarly, individuals aged 30–39 (AOR = 1.59, 95% CI = 1.22–2.08), 40–49 (AOR = 1.80, 95% CI = 1.39–2.35), 50–59 (AOR = 1.97, 95% CI = 1.54–2.52), and ≥ 60 years (AOR = 1.83, 95% CI = 1.42–2.36) had greater odds of being aware of HPC. Compared with individuals with six or less of education years, those with 7 to 12 years of education (AOR = 1.28, 95% CI = 1.03–1.59) and 13 years or more (AOR = 1.34, 95% CI = 1.05–1.71) showed greater odds of being aware of ACP. Similarly, those with 7 to 12 years of education (AOR = 1.73, 95% CI = 1.38–2.17), 13 or more years of education (AOR = 2.08, 95% CI = 1.61–2.67) showed greater odds of being aware of HPC. Compared to the first income quintile, the third income quintile showed greater odds (AOR = 1.22, 95% CI = 1.06–1.41), whereas the fifth income quintile showed lower odds (AOR = 0.71, 95% CI = 0.61–0.83) of being aware of ACP. Similarly, the third income quintile showed greater odds (AOR = 1.19, 95% CI = 1.02–1.37), whereas the fifth income quintile showed lower odds (AOR = 0.61, 95% CI = 0.52–0.71) of being aware of HPC. Compared with individuals who responded that their health was good, those responded that their health was poor showed lower odds of being aware of ACP (AOR = 0.61, 95% CI = 0.54–0.69) and HPC (AOR = 0.61, 95% CI = 0.55–0.69). Those who had recent outpatient visit showed greater odds of being aware of HPC (AOR = 1.34, 95% CI = 1.22–1.47). Hospitalization experience showed greater odds of being aware of ACP (AOR = 1.78, 95% CI = 1.29–2.46) and HPC (AOR = 1.74, 95% CI = 1.26–2.41). In addition, health checkup experience showed greater odds of being aware of ACP (AOR = 1.21, 95% CI = 1.10–1.32) and HPC (OR = 1.13, 95% CI = 1.03–1.24). Individuals who reported inadequate access to health care due to cost burden had lower odds of being aware of ACP (AOR = 0.50, 95% CI = 0.42–0.59) and HPC (AOR = 0.57, 95% CI = 0.48–0.67) compared with those who did not report inadequate access to health care due to cost burden. Those who had trust in the health care system showed greater odds of being aware of ACP (AOR = 1.86, 95% CI = 1.69–2.04) and HPC (AOR = 1.49, 95% CI = 1.36–1.64).

**Table 3:** Factors associated with awareness of advance care planning and hospice palliative care by multivariable logistic regression analysis (n = 13,546, N = 45.7)

Factors	Advance care planning		Hospice palliative care	
	OR (95% CI)	AOR (95% CI) ^a^	OR (95% CI)	AOR (95% CI) ^a^
Gender				
Male	1	1	1	1
Female	0.90 (0.83–0.98)*	0.92 (0.84–1.00)*	0.95 (0.88–1.03)	0.97 (0.89–1.06)
Age (years)				
15–19	1	1	1	1
20–29	1.12 (0.87–1.43)	1.07 (0.82–1.38)	1.29 (1.01–1.65)*	1.16 (0.89–1.51)
30–39	1.91 (1.50–2.43)**	1.76 (1.35–2.30)**	1.89 (1.48–2.41)**	1.59 (1.22–2.08)**
40–49	2.17 (1.71–2.77)**	1.97 (1.51–2.55)**	2.22 (1.74–2.84)**	1.80 (1.39–2.35)**
50–59	2.34 (1.86–2.96)**	2.31 (1.80–2.95)**	2.16 (1.71–2.74)**	1.97 (1.54–2.52)**
≥ 60	1.74 (1.38–2.19)**	1.99 (1.55–2.55)**	1.71 (1.35–2.16)**	1.83 (1.42–2.36)**
Education				
≤ 6	1	1	1	1
7–12	1.44 (1.19–1.74)**	1.28 (1.03–1.59)*	1.80 (1.47–2.20)**	1.73 (1.38–2.17)**
≥ 13	1.50 (1.23–1.81)**	1.34 (1.05–1.71)*	2.01 (1.64–2.46)**	2.08 (1.61–2.67)**
Household income quintile				
1st (lowest)	1	1	1	1
2nd	1.20 (1.06–1.36)**	1.11 (0.97–1.28)	1.30 (1.15–1.48)**	1.14 (0.99–1.31)
3rd	1.34 (1.18–1.52)**	1.22 (1.06–1.41)**	1.42 (1.25–1.60)**	1.19 (1.02–1.37)*
4th	1.17 (1.03–1.32)*	1.04 (0.90–1.20)	1.17 (1.03–1.33)*	0.95 (0.82–1.11)
5th (highest)	0.78 (0.68–0.89)**	0.71 (0.61–0.83)**	0.75 (0.66–0.86)**	0.61 (0.52–0.71)**
Self-rated health				
Good	1	1	1	1
Poor	0.71 (0.64–0.78)**	0.61 (0.54–0.69)**	0.70 (0.63–0.77)**	0.61 (0.55–0.69)**
Purchase of supplemental private health insurance				
No	1	1	1	1
Yes	1.06 (0.97–1.16)	0.93 (0.83–1.03)	1.12 (1.02–1.23)*	0.99 (0.89–1.11)
Recent outpatient visit				
No	1	1	1	1
Yes	1.02 (0.94–1.10)	1.01 (0.92–1.11)	1.23 (1.14–1.34)**	1.34 (1.22–1.47)**
Hospitalization in the past year				
No	1	1	1	1
Yes	1.54 (1.14–2.09)**	1.78 (1.29–2.46)**	1.36 (1.00–1.85)	1.74 (1.26–2.41)**
Health checkup in the past year				
No	1	1	1	1
Yes	1.27 (1.18–1.38)**	1.21 (1.10–1.32)**	1.22 (1.13–1.32)**	1.13 (1.03–1.24)**
Inadequate access to health care due to cost burden in the past year				
No	1	1	1	1
Yes	0.44 (0.38–0.52)**	0.50 (0.42–0.59)**	0.51 (0.43–0.60)**	0.57 (0.48–0.67)**
Trust in health care system				
Distrust	1	1	1	1
Trust	1.94 (1.77–2.12)**	1.86 (1.69–2.04)**	1.57 (1.43–1.72)**	1.49 (1.36–1.64)**

n, unweighted sample size; N, weighted sample size in millions; OR, odds ratio; AOR, adjusted odds ratio; CI, confidence interval

*, *p* < 0.05; **, *p* < 0.01

^a^Adjusted for gender, age, education, household income, self-rated health, purchase of supplemental private health insurance, experience of recent outpatient visit, experience of hospitalization and health checkup, inadequate access to healthcare due to cost burden, and trust in health care system

### Relationship between specific experiences of outpatient visits and awareness of ACP and HPC

A subgroup analysis was performed for participants who had a recent outpatient visit. Results of multivariable logistic regression analysis to examine the relationship between specific experiences of outpatient visits and awareness of ACP and HPC are presented in Table 4. Participants who visited a medical institution as a regular source of care had greater odds of being aware of ACP (AOR = 1.35, 95% CI = 1.12–1.62) than those who did not. Those who had sufficient conversation with the physician during outpatient visit showed greater odds of being aware of ACP (AOR = 1.25, 95% CI = 1.09–1.45) and HPC (AOR = 1.50, 95% CI = 1.29–1.73) than those who did not. Involving in decisions about their care and treatment had no significant relationship with awareness of either ACP or HPC.

**Table 4:** Associations between specific experiences during outpatient visit and awareness of ACP and HPC by multivariable logistic regression analysis (n = 7,782, N = 24.7)

Factors	Advance care planning		Hospice palliative care	
	OR (95% CI)	AOR (95% CI) ^a^	OR (95% CI)	AOR (95% CI) ^a^
Regular source of care				
No	1	1	1	1
Yes	1.33 (1.12–1.59)**	1.35 (1.12–1.62)**	1.04 (0.88–1.24)	1.08 (0.90–1.29)
Having sufficient conversation with physician				
No	1	1	1	1
Yes	1.34 (1.17–1.54)**	1.25 (1.09–1.45)**	1.58 (1.37–1.82)**	1.50 (1.29–1.73)**
Involving in decisions about care and treatment				
No	1	1	1	1
Yes	1.19 (1.00–1.41)	1.11 (0.92–1.33)	1.08 (0.91–1.29)	1.02 (0.85–1.22)

n, unweighted sample size; N, weighted sample size in millions; OR, odds ratio; AOR, adjusted odds ratio; CI, confidence interval

*, *p* < 0.05; **, *p* < 0.01

^a^Adjusted for gender, age, education, household income, self-rated health, purchase of supplemental private health insurance, experience of hospitalization and health checkup, inadequate access to healthcare due to cost burden, and trust in health care system

## Discussion

In our study, 39.3% and 35.7% of participants responded that they were aware of ACP and HPC, respectively. Comparing awareness across countries is challenging because of differences in health care system, legal implementation, and public insurance coverage. Nevertheless, ACP awareness investigated in our study was relatively lower than those of United States and European countries (40–90%) [34–36], but similar to or higher than those of Asian countries (3–36%) [37, 38]. Previous studies conducted prior to the enactment of the Act on Life-Sustaining Treatment Decisions found that Koreans’ awareness of ACP ranged from 10 to 16% [31, 37]. Results of the present study were based on the analysis of data after the new law was enforced, suggesting that public awareness might have improved compared to the past. Approximately 1.58 million AD forms and 105,000 POLST forms were registered in the four years after the law went into effect [29]. Such active participation of Koreans also supports our findings. Our finding is noteworthy in that ACP has recently been legislated. However, it is necessary to continuously investigate the awareness in future studies.

Awareness of HCP in our study was similar to that of a previous report regarding HPC awareness among Korean adults [27]. Compared to HPC awareness rates of 30–90% in other countries [9, 36, 39], the awareness rate in our study participants was relatively low. This result might be due to the relatively recent national policy to promote HPC and the low utilization of HPC compared to total deaths [24, 25, 27, 40]. Considering that the number of annual deaths continues to increase due to population aging, there is a need to increase public awareness of HPC and improve its use.

About half participants responded that they did not know either ACP or HPC. Less than a quarter of participants answered that they knew both ACP and HPC. A cultural reluctance to discuss death or serious medical conditions is still a barrier to EOL discussions between patients and providers in Korea [41]. Despite cultural barriers to talking about death, the public expects their health care providers to initiate EOL discussions at an appropriate time [31], not only when receiving emergency treatment or surgery for a critical illness [31], but also when receiving primary care for chronic diseases [42]. To improve public awareness of ACP and HPC, there is a need for healthcare providers to initiate more active discussions with the public and patients.

Our study described the association between public awareness of ACP and HPC and related factors among general population. We investigated sociodemographic characteristics, health status, health insurance, experience of health care services, and trust in the health care system.

Age, education, and household income were significantly associated with awareness of either ACP or HPC after controlling for all other variables. Results of our research were consistent with those of previous studies showing that older and highly educated people were more likely to be aware of ACP and HPC [36, 43, 44]. It is known that as people get older, they seek information to prepare for their EOL or loss of competence not only because they feel they are getting closer to death, but also because they have more experiences with death of loved ones [35]. A higher level of education correlates with a higher level of general health literacy, which can also extend to the context of preparing for EOL [45]. In our study, middle income was positively associated with awareness of either ACP or HPC, while high income was negatively associated with the awareness compared to low income. There are conflicting reports about how income affects awareness of ACP and HPC. Previous studies have reported that higher socioeconomic levels are related to higher awareness of ACP and HPC, indicating that health literacy could contribute to accurate knowledge [36, 46]. However, other research studies have shown that low income is positively correlated with HPC awareness, suggesting that people with low income are more willing to seek alternative options because of the high cost of curative treatment [45]. One study has also reported that income is not significantly related to awareness of ACP [19]. Our study showed that female participants were less likely to be aware of ACP than male participants, whereas gender had no significant relationship with awareness of HPC. Previous studies also have reported mixed results regarding gender. Some studies reported that females had higher awareness of ACP and HPC [36, 39, 47], whereas other studies revealed no significant associations between gender and such awareness [37, 43]. Further research is needed on the relationship between sociodemographic factors and awareness of ACP and HPC. Based on such research results, effective educational programs and campaigns must be developed to deliver targeted messages to different population groups.

Our study showed that chronic health conditions were not significantly related to awareness of ACP or HPC. In addition, participants who reported that their health status was poor were less likely to be aware of ACP and HPC than those reported that their health status was good. Prior research on the relationship between health status and awareness of ACP and HPC is mixed. Several studies have reported that diagnosis of chronic disease might reduce EOL discussion with doctors or affect non-accepting attitude toward EOL care planning [48, 49], which supports our finding and suggests that poor health itself can limit the ability to plan ahead or access to doctors. While some studies have reported that poor health condition is associated with positive attitude or willingness to implement ACP or HPC [38, 50, 51], other studies have shown that diagnoses of chronic diseases or self-rated health are not significantly associated with awareness of ACP or HPC [37, 47, 52, 53]. Due to mixed evidence from previous studies, further investigations are still needed to gain a clear understanding of the association between health status and awareness of ACP and HPC.

We investigated whether participants’ health insurance was related to awareness of ACP and HPC. Existing studies have reported that having health insurance is associated with greater awareness of ACP and positive attitude preference for HPC [51, 54–56]. However, the type of public health insurance or purchase of private health insurance was not significantly related to ACP or HPC awareness in our analysis. These results might vary depending on country-specific circumstances such as public health insurance schemes or insurance coverage for ACP and HPC services. The health care system of Korea is implemented under the National Health Insurance and Medical Aid program. All citizens are legally obliged to participate in it. These public insurance schemes cover the cost of ACP and HPC as well as treatment for serious conditions. Furthermore, many Koreans have voluntarily purchased supplemental private health insurance to alleviate financial burden of care not covered by the public health insurance [57]. Considering that Koreans are generally well-covered by public or private health insurance, the burden of healthcare costs may not have an impact as motivators to seek ACP and HPC.

Participants with experience of outpatient visit were more likely to be aware of HPC than those without. In those who had a recent outpatient visit, sufficient conversation with the physician during the visit was positively associated with awareness of either ACP or HPC. Regarding the medical institution as their regular source of care was also associated with awareness of ACP. Previous research findings have suggested that having a primary care provider is associated with awareness of ACP and HPC [53, 58]. Having a regular source of health care is also associated with adequate knowledge of HPC compared with having no such source [59]. Past studies have established that a primary care office visit is useful for ACP discussion because patients would prefer that their primary physician initiate the discussion [60]. Primary care physicians have information about patients in terms of their medical, psychological, and social background. Thus, they can assess patients’ capacity and needs regarding participation in ACP accurately [61]. Information obtained from health care providers can influence people’s intentions to use HPC early, which can ultimately result in positive quality of care [62].

Experience of hospitalization and health checkup was positively associated with awareness of either ACP or HPC. This result was consistent with results of previous studies showing that recent hospitalization was a significant predictor of ACP discussion [61], and HPC knowledge [59]. Previous research findings have also suggested that recent health care utilization such as treatment or screening is significantly related to ACP implementation [63, 64]. This suggests that patient experience as an intervention might be a mediator of patient and care provider interactions [61]. A prior study has also shown that people not only consider before all procedures or interventions with a high mortality risk, but also consider during hospitalization or health checkup as a suitable time for discussing ACP [31]. People prefer when they are relatively healthy to have their health care provider initiate a discussion about preparing for the future [65]. Another explanation for our results is that suffering from serious illness can influence decisions related to patient autonomy. In addition, receiving a diagnosis of certain diseases might trigger preparing for their future incapacity of decision-making [66, 67]. People tend not to think about preparing for the future until they realize they might definitely need it [64].

Among participants in our study, those who had experienced of inadequate access to health care due to cost burden were less likely to be aware of ACP and HPC compared to those who had not. This was consistent with results of prior research showing that perceived financial hardship to meet basic needs was associated poor knowledge about EOL practices, suggesting that it could be a result of reduced access to primary care [12]. Supporting this explanation, previous studies have shown that frequent exposure to the health care system is an important predictor of HPC knowledge [59] and that successful implementation of ACP is associated with patient-provider interactions built over multiple visits [68].

Distrust in the health care system might influence one’s low acceptability of ACP [69] with less-favorable beliefs and attitude about HPC [70]. Similar to these results, participants’ trust in the health care system was associated high awareness of ACP and HPC in our analysis. Existing studies have mainly raised cultural and racial disparities among different populations as the reason for such low awareness or preference [69, 71, 72]. However, Korea is a relatively homogeneous society with different sociodemographic aspects from other countries. Possible explanation is that people tend not to initiate ACP if they feel uncertain about whether a decision will be respected in the future [31]. Mistrust of health care providers or the health care system also can lead to patients’ misconceptions of being unreasonably abandoned or treated poorly, which can make them suspicious of EOL discussions [73]. As further research on distrust considering the complexity of this phenomenon, it is necessary to investigate what factors in the health care system cause mistrust. Through such studies, effective educational interventions and policy strategies can be deduced to dispel misconceptions about ACP and HPC by promoting trust. Participants with rigid attitudes toward the social system may give biased answers throughout the survey. While there was no evidence of a change in public trust in the health care system, it appears that awareness of ACP in Korea have changed significantly in a short period of time [31, 37]. Therefore, it can be assumed that the overall attitude toward the health care system of the participants in this study was less likely to distort their answers to the questionnaire. This is also supported by that the trust in the health care system in each country, as investigated in previous studies [74], differs from the international awareness of ACP or HPC [34–38].

Our study showed that experience of health care was closely related to the awareness of ACP and HPC. This suggests that the role of health care providers is important. Previous research studies have shown that the public is not only looking to health care providers as most preferred and reliable source of information, but also as enabler of discussions about ACP and HPC [60, 73, 75–77]. Given their accessibility and acceptability to the public, health care providers can increase public awareness by providing information about benefits and use of ACP and HPC [59]. People generally want health care providers to start the conversation about ACP and HPC, even agreeing that it is mandatory when they are diagnosed with a serious illness [49, 76]. However, such discussions with health care providers are lacking despite people’s wishes [42, 60, 78]. Health care providers might hesitate to initiate discussions because they believe patients will ask for information when they wish, or because they lack sufficient awareness/knowledge about ACP and HPC [76, 79]. There is a need to encourage health care providers to provide information and initiate discussions about ACP and HPC for healthy people as well as patients at risk of morbid events. This requires sufficient training of health care providers so that they can engage in culturally appropriate conversation. In addition, further research is needed on health-related factors so that health care providers can predict people’s capacity in order to initiate these discussions effectively [80].

To the best our knowledge, this is the first study that investigates public awareness of ACP and HPC among Korean after the implementation of a new law integrating the existing regulations on HPC with new regulations on ACP. This study particularly focused on the association between awareness and experience of healthcare services. In addition, a specific analysis was conducted for a group of people with outpatient visit experience who had hardly been investigated in previous studies. The current findings provide insights that raise awareness of ACP and HPC in terms of which health care is delivered and who provides the information. Our study provides current estimates of awareness of ACP and HPC and its influencing factors in a nationally representative sample based on population census data. Survey data were collected through one-on-one interviews by trained surveyors. Thus, our results are more generalizable to the general population than results of other studies that used convenience sampling method or analyzed self-reported survey data.

This study has limitations. First, variables such as experience with ACP or HPC through family or friends was not assessed because it was unavailable in the survey data. Prior research studies have revealed that experience with ACP and HPC through family, friends, and/or acquaintances is positively associated with the awareness [39, 58]. Close friends or relatives who have received HPC can be key sources of information of care [81]. Furthermore, being engaged with decision making for a family member with serious illnesses can be a mediator to acquire knowledge about ACP and HPC [58]. Second, this study was cross-sectional. Therefore, it was not possible to infer causal relationships. Third, since self-reported awareness of ACP and HPC was measured with Likert scales, limiting our understanding of the actual knowledge in the general public. More comprehensive measures of knowledge using multi-item scales are needed to distinguish accurate perception from possible misconceptions. Lastly, selection bias may have occurred because some targeted households did not participate in the survey and had to be replaced due to reasons such as absence or refusal. However, as a survey protocol to ensure the national representativeness of the sample, methods such as visiting targeted households multiple times or scheduling repeat visits were used.

## Conclusion

Our findings revealed a lack of awareness about ACP and HPC among Korean largely affected by their experience of health care. In addition, sociodemographic characteristics, health status, and trust in the health care system were independently associated with the awareness of ACP and HPC. Our findings indicate a crucial role for health care providers in providing information about ACP and HPC. Healthcare providers can initiate the conversation about ACP and HPC in an informative way in appropriate contexts, which might lead to less burdensome discussion than expected.

## Data Availability

Datasets from the Medical Service Experience Survey database are available for researchers who meet the accessibility criteria for obtaining confidential data. Researchers can apply for data on the Health and Welfare Data Portal operated by the Korea Institute for Health and Social Affairs (https://data.kihasa.re.kr).

## Abbreviations

ACP: Advance Care Planning
AD: Advance Directives
EOL: End of Life
HCP: Hospice Palliative Care
POLST: Physician Orders for Life-sustaining Treatment
